# Gender and chronic kidney disease in ankylosing spondylitis: a single-center retrospectively study

**DOI:** 10.1186/s12882-019-1658-6

**Published:** 2019-12-09

**Authors:** Wenling Ye, Jing Zhuang, Yang Yu, Hang Li, Xiaomei Leng, Jun Qian, Yan Qin, Limeng Chen, Xue-mei Li

**Affiliations:** 1 Department of Nephrology, Peking Union Medical College Hospital, Chinese Academy of Medical Sciences & Peking Union Medical College, Shuifuyuan 1, Wangfujing, Beijing, 100730 China; 2 Department of Nephrology, People’s Hospital of Xinjiang, Uygur Autonomous Region, 830001 China; 3 Department of Rheumatology and Clinical Immunology, Peking Union Medical College Hospital, Chinese Academy of Medical Sciences & Peking Union Medical College, Beijing, 100730 China; 4 Department of Orthopedics, Peking Union Medical College Hospital, Chinese Academy of Medical Sciences & Peking Union Medical College, Beijing, 100730 China

**Keywords:** Ankylosing spondylitis, Chronic kidney disease, Gender difference, Hyperuricemia, Hypertension

## Abstract

**Background:**

Ankylosing spondylitis (AS) is a well-known male-predominant inflammatory disease. This study aimed to assess the gender disparity in chronic kidney disease (CKD) in AS patients in China.

**Methods:**

AS patients were retrospectively studied at Peking Union Medical College hospital between January 2002 and June 2018.

**Results:**

Among 616 patients with AS, 154 (25.0%) patients had CKD (age, 41.8 ± 14.2 years; male:female, 3.2:1). Overall, 80 (13.0%) patients had only microscopic hematuria, 62 (10.1%) had proteinuria with or without hematuria, and 33 (5.4%) exhibited a reduced estimated glomerular filtration rate (eGFR, ≤60 mL/min/1.73 m^2^). Male CKD patients had more frequent proteinuria (*p* < 0.01), less microscopic hematuria only (*p* < 0.01), and lower eGFR (*p* = 0.04) compared with females. CKD was independently associated with hyperuricemia and total cholesterol in females, and with hyperuricemia, hypertension, and serum albumin in males. After follow-up for 1–7 years, five patients required renal replacement therapy including two patients who were already at stage 5 CKD when enrolled and three patients whose creatinine doubled. One patient died in the male group. No patients in the female group showed progression of renal dysfunction.

**Conclusions:**

CKD is a common comorbidity in patients with AS. Male patients are more likely to develop severe manifestations compared with female patients. Hyperuricemia was a strong independent risk factor for CKD in both genders, while hypertension and low serum albumin were risk factors for CKD only in males.

## Background

Ankylosing spondylitis (AS) is a chronic progressive inflammatory disease that primarily involves the spine and sacroiliac joints. The prevalence of AS was estimated to be 0.24% in Europe, 0.32% in North America, and 0.17% in Asia [1]. AS has been widely accepted as a male-predominant disease with a male-to-female ratio that was estimated to be as high as 9–10 in early studies [2, 3], and it ranges between 3.2:1 and 6.1:1 in most studies [4–8]. Studies have shown that there might be gender-attributable differences in patients with AS regarding disease characteristics, clinical outcomes, and radiographic damage, as well as in the response to treatment. Male patients are more likely to have a younger age of disease onset, more typical features, more severe and faster progression of radiographic structural damage, more common anterior uveitis, and a higher frequency of human leukocyte antigen B27 (HLA-B27) compared with female patients [5, 7, 9–12]. Females with AS show more active disease, less peripheral arthritis and functional disability as well as less psoriasis and a relatively worse treatment response with respect to both disease activity and functional outcome [6, 7, 9, 11, 13, 14].

AS can involve multiple systems and cause severe damage, especially the kidney, which is one of the systems commonly affected by AS. Several case series indicated the prevalence rate of renal involvement in AS is approximately 8.0 to 21.7% [4, 8, 15]. Renal involvement in patients with AS has been attributed to multiple factors, including the use of nephrotoxic medication, the presence of comorbidities such as hypertension, and complications [4, 16, 17]. We speculate that there may be a gender difference regarding involvement of the kidney in AS. However, few studies have investigated the gender disparity of CKD manifestation and prognosis or have focused on the risk factors for CKD in AS. Therefore, this study was designed to evaluate the possible contribution of gender to differences in clinal features, disease progression, and risk factors of CKD in AS.

## Methods

### Patient selection

This retrospective cohort study was conducted using patient-level data at the Peking Union Medical College Hospital between January 2012 and May 2018. All diagnoses of AS were confirmed by a rheumatological specialist. Inclusion criteria included: (1) patients who fulfilled the New York diagnostic criteria for AS established by the American College of Rheumatology (ACR) in 1984 [18]; (2) no signs of systemic lupus erythematosus, anti-neutrophil cytoplasmic antibody (ANCA)-associated vasculitis, acute infectious diseases, or malignant diseases; and (3) patients who were over 18 years old.

Baseline information was obtained from the patient’s first hospitalization. Detailed information regarding peripheral arthritis, extra-articular manifestations, presence of comorbidities, medications used for AS, physical examination and laboratory test results were recorded. Extra-articular manifestations and comorbidities included inflammatory bowel disease (IBD), uveitis, hypertension, diabetes, and kidney stones. History or current medications mainly included non-steroidal anti-inflammatory drugs (NSAIDs), disease-modifying anti-rheumatic drugs (DMARDs), or anti-tumor necrosis factor drugs (anti-TNFs). Laboratory tests included hemoglobin, routine urinalysis and 24-h urine protein, serum creatinine, uric acid, albumin, alanine transaminase (ALT) and lipid profile. Serum creatinine was tested by enzymatic methods. For human leukocyte antigen B27 (HLA-B27), only the tests performed at our hospital were analyzed. Acute-phase reactants included erythrocyte sedimentation rate (ESR) and C-reactive protein (CRP) levels. The estimated glomerular filtration rate (eGFR) was calculated based on the equation developed by the CKD–Epidemiology collaboration group (CKD-EPI) [19].

Patients were followed-up until June 2019 or until the primary outcome was reached. Every year follow-up information including serum creatinine, albumin, proteinuria, and whether renal replacement was initiated, was recorded since the first hospitalization. The composite primary outcome consisted of all-cause death, doubling of baseline serum creatinine, and/or dialysis initiation.

### Definitions

CKD was defined based on the Kidney Disease Outcomes Quality Initiative (K/DOQI) guidelines when patients had one of following abnormalities for 3 months or more [20]: (1) proteinuria; (2) microscopic hematuria; or (3) estimated glomerular filtration rate (eGFR) < 60 mL/min/1.73 m^2^. Proteinuria was considered to be either of at least grade 1+ by semiquantitative tests for urine albumin or proteinuria greater than 150 mg/24 h. Hematuria was defined as the presence of more than three red blood cells at a high magnification of the urinary sediment, or more than 80 red cells per microliter. Patients with hematuria underwent urinary sediment analysis to screen for dysmorphic erythrocytes. Nephrotic syndrome was defined as proteinuria greater than 3.5 g/24 h and a serum albumin level < 30 g/L. CKD was classified into five stages based on the K/DIGO Clinical Practice classification [20]: stage 1, eGFR 90–120 mL/min/1.73 m^2^; stage 2, eGFR 60–89 mL/min/1.73 m^2^; stage 3, eGFR 30–59 mL/min/1.73 m^2^; stage 4, eGFR 15–29 mL/min/1.73 m^2^; and stage 5, eGFR < 15 mL/min/1.73 m^2^. Renal dysfunction was defined as eGFR < 60 mL/min.1.73 m^2^. End-stage renal disease (ESRD) was defined as an eGFR < 15 mL/min/1.73 m^2^ or the need to start dialysis. Hypertension was defined as receiving antihypertensive medications, or systolic blood pressure ≥ 140 mmHg, or diastolic blood pressure ≥ 90 mmHg. Hyperuricemia was defined as serum uric acid level > 416 μmol/L in males, > 357 μmol/L in females, or a history of hyperuricemia [21].

### Statistical analysis

Statistical analysis was performed using SPSS version 21.0 (IBM, Armonk, NY, USA). Continuous variables with a normal distribution were expressed as the mean ± standard deviation (SD) and analyzed using the independent-sample *t*-test. Data with a non-normal distribution were expressed as the median and interquartile range and compared using the non-parametric Mann–Whitney *U*-test. The chi-square test was used to assess categorical variables. Multivariate logistic regression models with CKD or non-CKD as dependent variables were constructed to assess risk factors associated with CKD in two genders separately. Age, duration of AS, HLA-B27 positivity, the presence of hyperuricemia, hypertension, diabetes or kidney stones, ESR, CRP, serum albumin, triglyceride and total cholesterol were introduced into the multiple logistic regression model and analyzed by a stepwise process. Kaplan–Meier curves were constructed for primary outcome over time. The few missing data were supplemented by mean values for normal distribution variables or by the median for non-normal distribution variables. HLA-B27 was tested in approximately two thirds of patients in our hospital. The missing values of HLA-B27 were randomly treated with gender-specific ratios in this study. A two-sided value of *p* < 0.05 was considered statistically significant for all analyses.

## Results

### Characteristics of patients with ankylosing spondylitis

The clinical characteristics of patients with AS are shown in Table 1. Overall, 616 patients were enrolled in this study. There were 468 male patients and 148 female patients with a male-to-female ratio of 3.2:1. The mean age of the patients was 41.8 ± 14.2 years, and the mean duration since AS diagnosis was 12.6 ± 9.8 years. Positivity for HLA-B27 was observed in 87.7% of the patients. The prevalence of hyperuricemia and hypertension was 22.4% (138 patients) and 19.3% (119 patients), respectively. Among them, six (1.0%) patients had a history of gout attacks.

**Table 1:** Demographic and clinical features of patients with ankylosing spondylitis

Variable	All patients (*n* = 616)
Male, n (%)	468 (76.0)
Age, years	41.8 ± 14.2
Duration of AS, years	10.0 (5.0, 19.0)
Peripheral arthritis, n (%)	300 (48.7)
IBD, n (%)	23 (3.7)
Uveitis, n (%)	68 (11.0)
Hyperuricemia, n (%)	119 (19.3)
Diabetes mellitus, n (%)	53 (8.6)
Hypertension, n (%)	138 (22.4)
Kidney stone, n (%)	24 (3.9)
Medication for AS	
NSAIDs, n (%)	323 (52.4)
DMARDs, n (%)	270 (43.8)
Anti-TNFs, n (%)	72 (11.7)
BMI, kg/m^2^	23.5 ± 4.4
Systolic BP, mmHg	125.2 ± 18.2
Diastolic BP, mmHg	77.2 ± 11.9
Albumin, g/L	40.0 ± 5.7
Total cholesterol, mmol/L	4.3 ± 1.3
Triglyceride, mmol/L	1.3 (0.8, 1.6)
Uric acid, μmol/L	336.1 ± 111.3
ESR, mm/h	19.0 (12.0, 34.0)
CRP, mg/dL	10.2 (3.1, 28.1)
Serum creatinine, μmol/L	67.0 (57.0, 77.0)
eGFR, mL/min/1.73 m^2^	106.8 ± 22.5
HLA-B27 positive, n (%)	539 (87.5)

*Abbreviations: AS* ankylosing spondylitis, *IBD* inflammatory bowel disease, *NSAIDs* non-steroidal anti-inflammatory drugs, *DMARDs* disease-modifying anti-rheumatic drugs, *TNF* tumor necrosis factor, *BMI* body mass index, *BP* blood pressure, *ALT* alanine transaminase, *ESR* erythrocyte sedimentation rate, *CRP* C-reactive protein, *eGFR* estimated glomerular filtration rate, *HLA-B27* human leukocyte antigen B27

### Gender comparison of chronic kidney disease in ankylosing spondylitis

There were 154 (25.0%) AS patients with CKD. Among them, 59 (9.6%) patients presented with hematuria only and 80 (13.0%) patients had proteinuria with or without hematuria (Table 2). Episodic gross hematuria occurred in five (0.8%) patients and nephrotic syndrome in 11 (1.8%) patients. Renal dysfunction was observed in 33 (5.4%) patients.

**Table 2:** The prevalence and clinical manifestations of chronic kidney disease in ankylosing spondylitis

	All patients (*n* = 616)	Males (*n* = 468)	Females (*n* = 148)	*p* value
CKD, n (%)	154 (25.0)	119 (25.4)	35 (23.6)	0.74
Clinical manifestations				
Hematuria only, n (%)	59 (9.6)	35 (7.5)	24 (16.2)	< 0.01
Proteinuria, n (%)	80 (13.0)	72 (15.4)	8 (5.4)	< 0.01
eGFR ≤60 mL/min/1.73 m^2^, n (%)	33 (5.4)	29 (6.2)	4 (2.7)	0.15
CKD stages^a^				
1	100 (64.9%)	72 (60.5%)	28 (80.0%)	
2	21 (13.6%)	18 (15.1%)	3 (8.6%)	
3	27 (17.5%)	24 (20.2%)	3 (8.6%)	
4	4 (2.6%)	3 (2.5%)	1 (2.9%)	
5	2 (1.3%)	2 (1.7%)	0 (0.0%)	

*Abbreviations: CKD* chronic kidney disease, *eGFR* estimated glomerular filtration rate

^a^Patients with chronic kidney disease were used as the denominator to calculate the percentage

Compared with female CKD patients, male CKD patients had less frequent hematuria, more common proteinuria, and lower eGFR (Tables 2 and 3). The prevalence of renal dysfunction was 6.2 and 2.7%, respectively, in males and females, and two patients in the male group were already in ESRD. Additionally, male CKD patients were younger at AS onset and had a higher body mass index (BMI), more frequent hypertension, and a higher level of uric acid compared with females (Table 3). HLA positivity was more frequent in males compared with females. AS duration, IgA levels, ESR, and CRP were not significantly different between male and female patients.

**Table 3:** Comparison of clinical characteristics of CKD and non-CKD patients between genders

Variable	Females			Males			*p* value of female vs male CKD
	non-CKD (*n* = 113)	CKD (*n* = 35)	*p* value	non-CKD (*n* = 349)	CKD (*n* = 119)	*p* value	
Age, years	43.0 ± 13.3	46.5 ± 13.7	0.18	41.1 ± 13.9	41.3 ± 15.7	0.89	0.08
Age at AS onset, years	31.7 ± 14.1	35.8 ± 11.4	0.13	27.7 ± 13.3	28.0 ± 12.5	0.86	< 0.01
Duration of AS, years	10.0 (5.0, 16.0)	6.0 (3.0, 18.0)	0.64	12.0 (6.0, 20.0)	10.0 (5,0, 19.0)	0.20	0.39
Peripheral arthritis, n (%)	47 (41.6)	14 (40.0)	1.00	181 (51.9)	58 (48.7)	0.60	0.44
Hyperuricemia, n (%)	5 (4.4)	7 (20.0)	0.01	63 (18.1)	44 (37.0)	< 0.01	0.07
Hypertension, n (%)	19 (16.8)	6 (17.1)	1.00	68 (19.5)	45 (37.8)	< 0.01	0.03
Diabetes mellitus, n (%)	8 (7.1)	2 (5.7)	1.00	29 (8.3)	14 (11.8)	0.27	0.53
Kidney stone, n (%)	0 (0.0)	0 (0.0)	–	14 (4.0)	10 (8.4)	0.09	0.12
HLA-B27 positivity, n (%)	88 (77.9)	27 (77.1)	0.93	310 (88.8)	114 (95.8)	0.02	< 0.01
Medication use							
NSAIDs, n (%)	61 (54.0)	15 (42.9)	0.33	190 (54.4)	57 (47.9)	0.34	0.70
DMARDs, n (%)	50 (44.2)	18 (51.4)	0.56	154 (44.1)	48 (40.3)	0.52	0.25
Anti-TNF-α, n (%)	10 (8.8)	5 (14.3)	0.35	45 (12.9)	12 (10.1)	0.52	0.54
eGFR, mL/min/1.73 m^2^	111.4 ± 15.2	100.8 ± 25.7	0.03	111.7 ± 16.0	89.8 ± 32.8	< 0.01	0.04
Serum creatinine, μmol/L	53.0 (47.0, 59.0)	56.0 (46.0, 67.0)	0.12	69.0 (62.0, 75.0)	80 (70.0, 123.0)	< 0.01	< 0.01
Albumin, g/L	41.1 ± 4.8	39.2 ± 6.6	0.11	40.7 ± 5.0	37.3 ± 7.1	< 0.01	0.16
Total cholesterol, mmol/L	4.3 ± 1.1	4.8 ± 1.1	0.04	4.1 ± 1.0	4.6 ± 2.0	0.04	0.55
Triglyceride, mmol/L	1.3 (0.8, 1.4)	1.4 (0.8, 1.4)	0.17	1.2 (0.8, 1.4)	1.5 (0.8, 1.8)	< 0.01	0.37
CRP, mg/dL	6.1 (1.9, 19.9)	8.6 (1.8, 38.9)	0.29	12.5 (4.0, 32.0)	12.6 (3.2, 26.8)	0.36	0.67
ESR, mm/h	23.0 (18.0, 32.0)	28.0 (19.0, 37.0)	0.10	17.0 (11.0, 31.0)	23.0 (14.0, 44.0)	< 0.01	0.31

*Abbreviations: AS* ankylosing spondylitis, *HLA-B27* human leukocyte antigen B27, *NSAIDs* non-steroidal anti-inflammatory drugs, *DMARDs* disease-modifying anti-rheumatic drugs, *TNF* tumor necrosis factor, *eGFR* estimated glomerular filtration rate, *CRP* c-reactive protein, *ESR* erythrocyte sedimentation rate

### The risk factors associated with chronic kidney disease in both genders

Compared with non-CKD patients with AS, male CKD patients had more common hyperuricemia and hypertension, higher total cholesterol and triglyceride levels, lower albumin levels, higher ESR level, and more common renal dysfunction (Table 3). In female patients, only the prevalence of hyperuricemia, total cholesterol level, and eGFR were significantly different between CKD and non-CKD patients.

Age, duration of AS, HLA-B27 positivity, the presence of hyperuricemia, hypertension, diabetes or kidney stones, ESR, CRP, serum albumin, triglyceride and total cholesterol, which might be associated with CKD development, were introduced into the multiple logistic regression model. They were analyzed by a forward stepwise process in female and male patients separately. Logistic regression showed that hyperuricemia, hypertension, high total cholesterol level, and low albumin were independent risk factors for CKD development in male patients. In female patients, only hyperuricemia and total cholesterol hypertension were independent factors that were associated with CKD (Table 4).

**Table 4:** Factors associated with CKD analyzed by logistic regression based on gender

Variable	Odds ratio (95% CI)	*p value*
Females		
Hyperuricemia (1 = yes)	5.14 (1.39, 19.04)	0.01
Total cholesterol, 1 mmol/L	2.17 (1.32, 3.58)	< 0.01
Males		
Hyperuricemia (1 = yes)	2.58 (1.57, 4.22)	< 0.01
Hypertension (1 = yes)	2.40 (1.48, 3.90)	< 0.01
Albumin, 1 g/L	0.90 (0.86, 0.93)	< 0.01

*Abbreviations: CI* confidence interval, CKD or non-CKD as dependent factor and age, duration of AS, HLA-B27 positivity, the presence of hyperuricemia, hypertension, diabetes or kidney stones, erythrocyte sedimentation rate, C-reactive protein, serum albumin, triglyceride, and total cholesterol as dependent factors

### Outcomes of chronic kidney disease in both genders

**Fig. 1:**
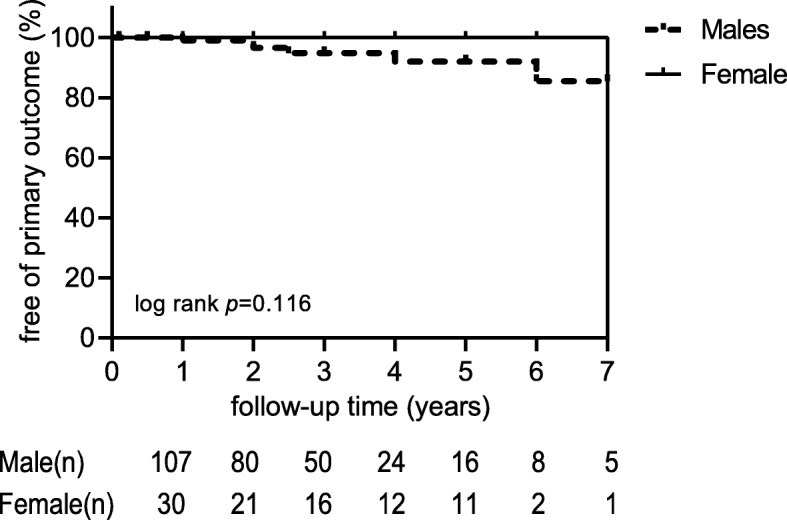
Primary outcome over time in male and female CKD patients. Primary outcome consisted of doubling of serum creatinine, dialysis initiation or all cause death

Excluding 15 patients who were lost to follow-up, the mean follow-up duration was 2.8 ± 1.6 years (range, 1–7 years). A Kaplan–Meier curve by gender category showed that gender was not significantly associated with the primary outcome (log rank test, *p* = 0.12) (Fig. 1). All patients with renal function decline were in the male group. Overall, seven patients in the male group reached the primary outcome after 3.0 years of follow-up, excluding two patients who were already on hemodialysis at the time of enrollment. Among the seven patients, three patients entered ESRD and received renal replacement therapy after 1.5–3 years, three patients showed a doubling of creatinine levels, and one patient whose renal creatinine was doubled before death died of pulmonary infection. In the female group, no primary outcome was observed after an average follow-up of 2.9 years.

## Discussion

AS, which is a form of spondyloarthritis, is a chronic, multisystemic inflammatory disease that primarily involves the spine and the sacroiliac joints. The most common extra-articular manifestations are uveitis, bowel disease, and heart, lung, and skin conditions. Renal involvement is also common in AS. In a population-based study from Canada, the occurrence of renal complications consisted of acute kidney injury, CKD, amyloidosis, and hypertensive renal disease, and they occurred in 3.4% of men and 2.1% of women with AS [22]. The present study showed a frequency of CKD in patients with AS of 25.0%. Compared with a prevalence of CKD from 9.2 to 10.8% in the Chinese general population [23, 24], this study showed that the prevalence of CKD is much higher in AS patients.

AS is considered a disease that occurs predominantly in males. In the present study, the male-to-female ratio was 3.2:1. Several studies have revealed that differences exist in clinical manifestation, age of disease onset, radiographic skeletal damage, medication response, and disease progression between males and females with AS [5–7, 9–14]. However, few published studies have investigated gender-attributable differences with respect to CKD in patients with AS. Understanding the gender-associated differences in disease progression and risk factors for CKD in AS are important to guide disease prevention strategies and further treatment in individuals with these coexisting conditions.

In the present study, clinical manifestations of CKD varied from microscopic hematuria and proteinuria to nephrotic syndrome, and from mild-renal dysfunction to ESRD. Proteinuria with or without hematuria was the most common presentation of renal involvement, affecting 13.0% of patients, while 9.6% patients presented with hematuria only and 5.4% patients developed renal dysfunction. Wu et al. reported that among the 926 AS patients, 21.7% patients had renal involvement, including 14.3% with hematuria, 4.8% with proteinuria, 0.1% with reduced eGFR, and 2.6% with multiple renal involvement manifestations [8]. In a retrospective single-center study from South Korea, 8% of AS patients had abnormal urinalysis test results as follows: proteinuria (5.9%), hematuria (2.8%), or both (0.7%) [4]. In our study, the prevalence of proteinuria and renal dysfunction were much higher compared with these two studies from Asia. However, compared with these two studies, patients enrolled in our study were older and had a longer duration of AS [4, 8]. These differences may explain the differences in CKD severity between the three studies. However, the differences in these results also indicate that the prevalence and severity of CKD may increase with the patient’s age and the duration of their disease.

We found that clinical manifestations of CKD showed gender disparity. Male CKD patients had less frequent hematuria (*p* < 0.01), more frequent proteinuria (*p* < 0.01), and a lower eGFR (*p* = 0.04). Although a Kaplan–Meier curve did not show a significant association between gender and CKD outcome, all patients with renal function decline were in the male group. However, in the female group, no primary outcome was observed after follow-up. These results suggested that renal involvement was worse in males compared with females. In previous studies [5, 7, 10], male patients had a younger age of AS onset, a higher BMI, more frequent occurrence of hypertension, and a higher level of uric acid compared with female patients in this study. Additionally, HLA positivity was more frequent in males compared with females.

Few studies have investigated the risk factors of CKD in AS. In a study that enrolled 681 patients, uric acid and IgA levels were significantly higher in patients with proteinuria compared with those without proteinuria [4]. An international, cross-sectional study that enrolled 2098 patients showed that age, HLA-B27 positivity, and CRP were independently associated with renal impairment (eGFR < 60 mL/min/1.73 m^2^) in patients with spondyloarthritis [25]. In this study, we used CKD to evaluate renal involvement including hematuria, proteinuria, and chronic renal dysfunction based on the K/DOQI criteria. To assess the possible disparity of risk factors for CKD in AS, we divided patients into CKD and non-CKD groups based on two genders (male and female). Male CKD patients had more frequent hyperuricemia, hypertension and HLA-B27 positivity, higher total cholesterol and triglyceride levels, lower albumin levels, higher ESR and more common renal dysfunction compared with non-CKD patients. A multivariate logistic regression analysis showed that hyperuricemia, hypertension, and serum albumin level were independent risk factors for the development of CKD in males. However, hyperuricemia and total cholesterol were independent factors for CKD in female patients. No differences in HLA-B27 positivity and AS disease activity were found between CKD and non-CKD groups in the two genders.

The present study showed that hyperuricemia had the strongest association with CKD in AS patients of both genders, showing that hyperuricemia may play an important role in the CKD development in this population. Although only 1.0% of patients experienced a gout attack, the prevalence of hyperuricemia was 19.3% overall and 33.1% in CKD patients. A previous study showed that uric acid was a strong correlate of renal dysfunction in RA patients [26]. Studies in the general population and in patients with diabetes or CKD also confirmed the association of hyperuricemia with the development and progression of kidney disease [27–29]. In two community-based cohorts involving 13,338 healthy participants who were followed for a mean of 8.5 years, every 1 mg/dL increase in baseline serum uric acid level was associated with a 7% increased risk for developing kidney disease (defined as an eGFR < 60 mL/min/1.73 m^2^) [28]. An investigation of 324 patients in the Coronary Artery Calcification in Type I Diabetes (CACTI) study showed that for every 1 mg/dL increase in uric acid at baseline, there was an 80% increased risk of developing micro- or macroalbuminuria [29]. Several underlying mechanisms have been proposed for these effects [30, 31]. Uric acid crystals can adhere to the surface of renal epithelial cells and induce tubulointerstitial inflammation [32]. Hyperuricemia had effects on glomerular hemodynamics by increasing renal vascular resistance [30]. Uric acid may directly affect endothelial smooth muscle cells in the vascular walls by blocking nitric oxide release, and inducing afferent renal arteriolopathy through effects on the renin-angiotensin-aldosterone system [33–36]. A clinical study confirmed these findings, showing that hyperuricemia was significantly associated with a higher risk of renal arteriolar hyalinosis and higher-grade wall thickening in patients with CKD [37]. Additionally, hyperuricemia is also associated with a greater risk of kidney stones, which have been shown to increase the risk of CKD and reduced eGFR [38]. In our study, kidney stones occurred in 3.9% of AS patients, but no association was observed between urolithiasis and CKD, suggesting that urolithiasis is not a main risk factor in this population.

The increasing prevalence of hyperuricemia appears to have various causes, including increased longevity, obesity, alcohol consumption, and a high dietary intake of meat and seafood [39]. Hyperuricemia may also be related to risk factors associated with medications that are used for AS or coexisting comorbidities. Several medications have been associated with the development of hyperuricemia by impairing uric acid excretion [40, 41]. DMARDs are widely used for second-line therapy in AS. Sulfasalazine is the most extensively studied DMARDs and more than 40% of patients received sulphasalazine treatment in the present study. Although sulphasalazine has not been shown to be associated with hyperuricemia, the pharmacokinetics of sulphasalazine were reviewed to identify any association with hyperuricemia [42]. Additionally, diuretics, low-dose aspirin, angiotensin converting enzyme inhibitors, and non-losartan angiotensin II receptor blockers were prescribed for some patients, including patients with hypertension, congestive heart failure, coronary heart disease, and stroke. Those medications may affect hyperuricemia to some degree. Additionally, hyperuricemia is the result of dysfunctional urate homeostasis. Approximately two-thirds of the uric acid produced in humans is excreted by the kidneys [43]. Renal impairment can further interfere with renal excretion of uric acid and aggravate hyperuricemia.

This study showed that hypertension was a common comorbidity in the AS population, and the overall prevalence of hypertension was 22.4% in AS patients. A previous study also showed that the prevalence of hypertension in patients with AS was more frequent compared with matched cohorts [44]. In our study, the prevalence of hypertension was significantly higher in male CKD patients compared with female CKD patients (37.8% vs 17.1%, *p* = 0.025) and multiple regression analysis showed that hypertension is a risk factor for CKD only for male patients. The potential mechanism involved is unclear although it might be related to the role of sex hormones. Observational studies suggest gender differences in the prevalence or outcome of AS, hypertension, and CKD [6, 45, 46]. Male hormones exert a deleterious effect in terms of activating the renin-angiotensin system (RAS), stimulating endothelin synthesis, and increasing oxidative stress, which are closely associated with hypertension and renal injury. Testosterone increased efferent arteriolar resistance by activating the RAS and increased angiotensin type 1 receptor expression in mesangial cells [45, 47]. Estrogen inhibits endothelin synthesis and restrains its vasoconstrictor effects, whereas testosterone has the opposite effect. Testosterone also increased oxidative stress by increasing the generation of reactive oxygen species and by inhibiting antioxidant enzymes to increase blood pressure and aggravate renal injury [45].

It is well-known that the occurrence of hypertension increases the risk of all renal complications. Several potential mechanisms that were studied in other conditions have been explored to examine the association between uric acid and hypertension. Hyperuricemia promotes the development of hypertension and induces a renal arteriolopathy by causing vascular smooth muscle cell proliferation and activating the RAS [35]. Clinical data also support a potential link between uric acid and hypertension. A recent study assessing the long-term efficacy of lowering uric acid with febuxostat on hypertension and renal function showed that lowering uric acid levels was an effective treatment for hypertension and CKD progression [48]. The causal relationship between hyperuricemia and hypertension in the development of CKD in AS requires further investigation.

HLA-B27 is the main genetic component of AS susceptibility. Its prevalence is lower among women with AS compared with men in several studies [5, 7, 13]. An international study investigating comorbidities in SpA showed that HLA-B27 positivity was significantly associated with renal impairment (eGFR < 60 mL/min/1.73 m^2^) [25]. In our study, HLA-B27 positivity was observed in 87.5% of patients and it was also more frequent in male CKD patients compared with male non-CKD patients (95.8% vs 77.1%, *p* < 0.01). However, HLA-B27 positivity did not independently correlate with the presence of CKD in both genders. The differing results between the two studies may be related to the patients’ ethnic backgrounds.

## Conclusions

Our findings suggest that there were some gender differences in the clinical manifestations and risk factors of CKD in patients with AS. The manifestations of renal involvement seem to be more severe in males compared with females, although the prevalence of CKD was similar. The presence of hyperuricemia was a strong predictor of renal involvement for both genders of AS patients, while hypertension and low serum albumin are specific predictors for CKD only in male patients. This study suggests that AS patients with elevated uric acid and hypertension, especially in males, may require screening for CKD and appropriate management.

There are some important limitations in this study. First, the prevalence of CKD may be overestimated compared with the general AS patient population. More severely affected patients who needed hospitalization were enrolled in this study, and therefore, this might have introduced selection bias. Second, the duration of follow-up (mean, 2.8 years) was not long enough to reach the primary outcome for most of the patients. A longer follow-up duration is warranted to further investigate the impact of gender on disease progression and renal prognosis in this population. Despite the limitations described above, this is the first study to focus on gender disparity in Chinese patients with AS and the findings presented here could help to elucidate the potential influence of gender on clinical features, disease severity, and risk factors for CKD in this population.

## Data Availability

The datasets used and analyzed during the current study are available from the corresponding author on reasonable request.

## Abbreviations

ALT: Alanine transaminase
anti-TNF: Anti-tumor necrosis factor
AS: Ankylosing spondylitis
CKD: Chronic kidney disease
CRP: C-reactive protein
DMARDs: Disease-modifying anti-rheumatic drugs
eGFR: Estimated glomerular filtration rate
ESR: Erythrocyte sedimentation rate
ESRD: End-stage renal disease
HLA-B27: Human leukocyte antigen B27
IBD: Inflammatory bowel disease
Ig: Immunoglobulin
K/DOQI: The Kidney Disease Outcomes Quality Initiative
NSAIDs: Non-steroidal anti-inflammatory drugs
